# Development and Implementation of MBR Monitoring: Use of 2D Fluorescence Spectroscopy

**DOI:** 10.3390/membranes12121218

**Published:** 2022-12-02

**Authors:** Claudia F. Galinha, João G. Crespo

**Affiliations:** LAQV-REQUIMTE, Chemistry Department, NOVA School of Science and Technology, Universidade NOVA de Lisboa, 2829-516 Caparica, Portugal

**Keywords:** membrane bioreactor (MBR), monitoring, 2D fluorescence spectroscopy, fluorescence EEMs, fouling, machine learning

## Abstract

The monitoring of a membrane bioreactor (MBR) requires the assessment of both biological and membrane performance. Additionally, the development of membrane fouling and the requirements for frequent membrane cleaning are still major concerns during MBR operation, requiring tight monitoring and system characterization. Transmembrane pressure is usually monitored online and allows following the evolution of membrane performance. However, it does not allow distinguishing the fouling mechanisms occurring in the system or predicting the future behavior of the membrane. The assessment of the biological medium requires manual sampling, and the analyses involve several steps that are labor-intensive, with low temporal resolution, preventing real-time monitoring. Two-dimensional fluorescence spectroscopy is a comprehensive technique, able to assess the system status at real-time without disturbing the biological system. It provides large sets of data (system fingerprints) from which meaningful information can be extracted. Nevertheless, mathematical data analysis (such as machine learning) is essential to properly extract the information contained in fluorescence spectra and correlate it with operating and performance parameters. The potential of 2D fluorescence spectroscopy as a process monitoring tool for MBRs is, therefore, discussed in the present work in view of the actual knowledge and the authors’ own experience in this field.

## 1. Monitoring of Membrane Bioreactors

Membrane bioreactors (MBRs) couple a biological reactor with a membrane, allowing the retention of solids and macromolecules (depending on membrane characteristics) during the biological reaction, while the fluid is permeated through the membrane, allowing for continuous use of the biological catalyst and retention of compounds according with their size. Due to the direct contact between the membrane and the highly complex biological media, MBRs are particularly vulnerable to the development of fouling caused by the adsorption of colloidal and soluble material at the membrane surface and pores, as well as to the adhesion/deposition of biomass (composed by cells, organic and inorganic compounds). 

Despite the large applicability of MBRs in different processes, they are mostly known and studied for wastewater treatment, offering several advantages, such as high effluent quality and stability (with potential for water reuse), retention of microbial species independent of hydraulic retention time, and reduced footprint. However, the application of MBRs is still conditioned by the inevitable membrane fouling, high costs associated with aeration, and complex control systems required. In fact, the mitigation of membrane fouling requires the use of operating strategies to limit fouling development (such as intermittent permeation and use of larger coarse bubbles to scour the membrane surface) and frequent membrane cleaning (with or without the use of chemicals).

In MBRs for wastewater treatment, monitoring is also essential to ensure the quality and stability of the permeated effluent and meet legal requirements for discharge. Monitoring the biological reaction is essential to characterize the influent and effluent streams, the biological media inside the reactor, and the biological activity. Additionally, the organic compounds present in the biological media, deriving both from the incoming wastewater and from the microbial activity, are generally grouped under the term of extracellular polymeric substances (EPS) and can be classified into bound EPS, when they are attached to cells to form biomass aggregates, and soluble EPS (also called soluble microbial products, SMP), when freely suspended in the media. EPS are generally assumed to be the major cause of fouling in MBRs [1]; therefore, monitoring their concentration and composition is considered essential.

Biological activity can be characterized by common parameters usually assessed in bioreactors, such as the amount of biosolids in the bioreactor (total suspended solids (TSS) and volatile suspended solids (VSS)), and the composition of feed, mixed liquor, and permeate. This characterization usually involves assessing the organic carbon (chemical oxygen demand—COD; biochemical oxygen demand—BOD (in 5 days: BOD_5_); total organic carbon—TOC), the presence of ionic species (nitrogen (ammonia, nitrite, nitrate, organic N, total N); phosphorus (total P, orthophosphate)), and other specific compounds, such as toxic compounds that may be present in wastewaters. Additionally, the dissolved oxygen, pH, and temperature are usually monitored, and then controlled if required. While some parameters are easily assessed online with probes (dissolved oxygen, pH, and temperature), and the presence of solids is actually becoming common to assess with specific online probes, the assessment of carbon, nitrogen, and phosphorus usually requires sampling and different preprocessing steps, e.g., filtration and digestion, prior to be analyzed (according with standard methods [2]).

Microbiologic assessments are also relevant, especially when applying mixed microbial cultures. In the biological treatment of wastewaters, it is essential to monitor the presence of both beneficial and hazardous microorganisms. In addition to the presence of virus and other pathogens in the effluent water, the analysis of specific groups of microorganisms (identification and abundance analysis of the microbial communities), able to degrade or accumulate compounds (e.g., toxic compounds, phosphorus), is often performed to assess the ability of the biomass to remove such compounds. However, the methods used for these assessments also rely on sample collection, and they often require microbial cultivation or DNA/RNA analysis, which are time-consuming and require specialized analysts. 

Membrane performance is assessed primarily through flux and transmembrane pressure. To achieve a continuous flow in MBRs, they are usually operated with imposed permeate flux while the transmembrane pressure (TMP) is monitored online [3]. This online measurement is essential and allows following the evolution of membrane permeance. However, through the evolution of transmembrane pressure (or of permeance), it is not possible to distinguish the fouling mechanisms occurring in the system or predict the future behavior of the membrane. In MBRs operated under controlled permeate flux, a two-step phenomenon is always noticed with a first stable or slow TMP increment followed by a sudden and sharp increase (called “TMP jump”). This behavior is related to the concept of critical flux, but is hard to predict, due to the several factors affecting fouling evolution, e.g., media composition, biomass characteristics, and activity. Critical flux is a concept defined as the flux at which there is a balance between the scour forces at the membrane surface (promoted by agitation, crossflow, or air bubbles) and the filtration forces, and it depends greatly on media characteristics and filtration setup and operation. MBR systems are usually operated at a sustainable flux, defined as a flux bellow the critical flux [4], to avoid the deposition of solids at the membrane surface due to pressure. This strategy increases the time of operation with a stable TMP; however, it does not fully prevent the TMP jump, due to the complexity of interactions between different compounds/microorganisms and the membrane surface. Therefore, various methods and techniques have been used for the characterization of foulants and monitoring fouling development. 

One of the most studied characteristics of biological media in MBRs for wastewater treatment is the concentration of biomass (suspended solids or mixed liquor suspended solids (MLSS)). The effect of suspended solids in permeance and fouling development is highly relevant as they directly impact the filtration process through deposition or adherence at membrane surface. However, the correlation between fouling and MLSS concentration can change for different biomass concentrations and filtration conditions (e.g., for MLSS < 6 g/L, fouling development was found to be inversely correlated with biomass concentration, but directly correlated for MLSS > 15 g/L [5]). Therefore, facing MLSS changes, an MBR may require adjustments of the permeate flux (i.e., control of flux to prevent sudden TMP rise) and application of different filtration strategies (e.g., intermittent permeation or backflush) to reduce the formation of a cake layer and maintain a sustainable flux [4,6].

Additionally, the presence and composition of EPS in media also affects the filtration and fouling formation in different ways according with the compounds’ size and physicochemical properties. Proteins and polysaccharides are the most abundant EPS components in wastewater treatment systems and are usually assumed to be the major contributors for fouling [3]; therefore, the evaluation of EPS concentration in MBRs relies mostly on the measurement of these two classes of compounds in the media (soluble EPS) or after extraction (from cells or from the membrane), through colorimetric methods (e.g., Lowry [7] and Dubois [8]). For the characterization of fouling agents, chromatographic methods have also been applied after a previous separation step (usually centrifugation and/or filtration). Size exclusion chromatography (SEC) is used to characterize EPS using the molecular size corresponding to polysaccharides, some proteins, colloids, and humic compounds (e.g., [9,10,11]). However, despite the detailed information that can be obtained about specific compounds in the MBR, such characterization techniques are not able to be used for real-time monitoring due to their technical requirements and limitations.

One of the most common ways to assess the fouling potential of the biological media is to perform filterability tests on the media (e.g., [12,13]) and/or use TMP to calculate membrane fouling rates and membrane resistances (e.g., [14,15,16]). Filterability tests can be performed by directly filtering the samples of media or filtering different extracts obtained from raw samples (e.g., using a dead-end module [12]). The data obtained from these tests and correlated with the fouling ability of media can be the permeance data of samples or the water permeance of the fouled filter after filtering a sample (through calculation of resistance). Capillary suction time (CST) is also used to assess sludge filterability (through dewaterability) and is easier to perform in a reproducible way using commercial equipment already available. One study compared the two methods using samples collected frequently from a MBR for wastewater treatment and concluded that the results are similar for both techniques, although CST is easier to perform for intensive monitoring, due to its simplicity [13]. This study also verified that the filterability of the biological media changes significantly during long-term operation, affecting fouling evolution.

Although the results from these analytical methods are not enough to predict a TMP jump, they are a good source of information about the media composition and characteristics, and filterability tests indicate the risk of a TMP jump occurring (when filterability is low).

In fact, three basic fouling factors were previously indicated for MBRs in wastewater treatment: (i) the nature of the biological media, (ii) the membrane properties, and (iii) the hydrodynamic environment experienced by the membrane [6]. Therefore, since membrane properties are set previously, monitoring an MBR requires not only the characterization of the biological media in contact with the membrane, but also the characterization of the chemical and physical events occurring at membrane surface.

The assessment of membrane rejection behavior also requires the characterization of the permeate and of the biological media. Variations of membrane selectivity along time are also an indicator of the membrane performance and may indicate the development of a fouling layer, membrane clogging, or membrane deterioration. 

Despite the numerous methods already studied and applied to characterize MBR systems, characterization is not the same as monitoring, as monitoring implies following the process development and performance over time. Monitoring (and control) requires methods that are able not only to characterize the systems, but also to find correlations between the analytical results and the process performance and/or fouling development. Therefore, several studies investigated the development of correlations and models (either mechanistic or based on artificial intelligence) for prediction of fouling development, membrane performance, and cleaning requirements (e.g., [17,18,19,20]). Independently of the mathematical methods used, all rely on accurate data from the MBR system, which is highly vulnerable to feed and biological variations. However, most of the characterization methods require sampling and analytical reagents, and they are time-consuming (it may take more than 1 day to know the results). Additionally, membrane cleaning with chemicals requires to stop the filtration process and take out or isolate the membrane module, which should be minimized.

Therefore, the development of novel monitoring and measurement approaches that are online and/or real-time are required for the optimization and control of the MBR process, in order to anticipate the upcoming of process anomalies and support decision making. This is especially important to support decisions about membrane cleaning and, eventually, the right moment for their replacement.

In this context, different spectroscopic methods have been explored not only for MBR monitoring, but also to monitor other biological and membrane processes, due to their noninvasive and reagent-free properties. The application of 2D fluorescence spectroscopy as a monitoring tool is derived from the possibility of using fluorescence in large ranges of excitation and emission wavelengths to simultaneously detect the presence of several natural fluorophores in biological media and cells. In fact, the first use of an entire fluorescence spectrum obtained by 2D fluorescence spectroscopy, without band selection, was proposed in 2001 to monitor an extractive MBR [21]. However, the use of the information contained in such fluorescence spectra is far from trivial, and different approaches can be used according to the monitoring objectives. According to the actual knowledge and the authors’ own experience in this field, the next sections provide an overview of 2D fluorescence spectroscopy’s applicability to characterize and monitor different aspects of MBRs, the methods used to extract information from fluorescence spectra, and future perspectives for monitoring and control based on 2D fluorescence spectroscopy.

## 2. Two-Dimensional Fluorescence Spectroscopy

### 2.1. Use of 2D Fluorescence Spectroscopy to Characterize MBR Systems

In MBR systems, particularly for wastewater treatment, the culture media have a complex composition, requiring the assessment of several compounds simultaneously. Additionally, noninvasive and nondestructive measuring systems are preferable to avoid disturbing the biological system and allow frequent assessment. Furthermore, despite the development of new sensors able to monitor various compounds, the increase in the number of monitoring parameters raises the costs of monitoring. Therefore, multivariate methods able to simultaneously detect several compounds/parameters become cost-effective. If those methods are applied online, they may also allow for real-time monitoring and control, including the ability to detect “accidents” such as the appearance of undesirable byproducts or the presence of toxic compounds. 

**Figure 1:**
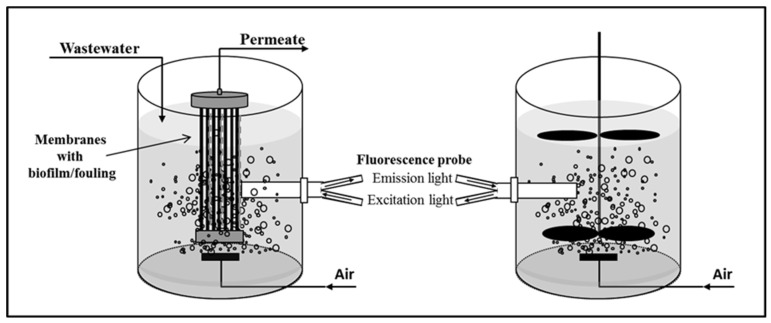
Acquisition of 2D fluorescence scans at membrane surface in submerged membrane bioreactors (**left**) and in bulk solution of bioreactors (**right**) using optical probes.

In view of such requirements, spectroscopic methods are good candidates to monitor the biological process in MBRs. Within several spectroscopic techniques available, fluorescence spectroscopy is able to detect fluorescent compounds naturally present in biological systems and in wastewater (e.g., NADH, some vitamins and cofactors, and organic compounds with delocalized electrons), regardless of being intra- or extracellular, most significantly the presence of potential fouling agents (proteins and humic acids). Additionally, the simultaneous scan of several excitation and emission wavelengths (two-dimensional (2D) fluorescence spectroscopy) allows assessing various fluorophores within one spectrum and can be performed online, directly in the media or at membrane surfaces, with an optical probe, without requiring sampling or disturbance of the MBR system (Figure 1). This fluorescence technique can then be used as a multiparameter monitoring tool in MBRs.

**Figure 2:**
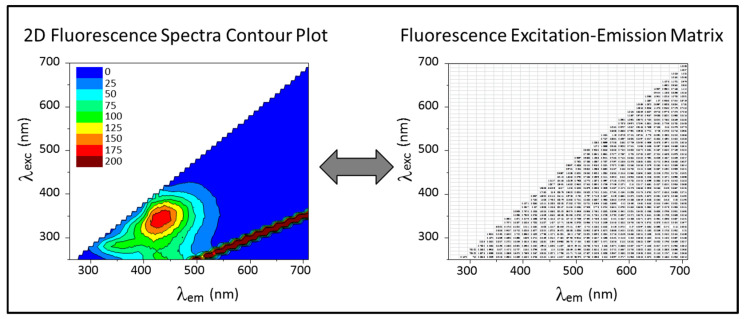
Two-dimensional fluorescence spectra represented as a contour plot (**left**) and as a numerical matrix (**right**).

When using 2D fluorescence spectroscopy, the samples are assessed by scanning a range of selected excitation/emission wavelengths, covering a wide spectral region. These fluorescence scans result in large matrices (excitation/emission matrices, EEMs), where the intensity of fluorescence emission is recorded for each pair of excitation (λ_ex_) and emission (λ_em_) wavelengths. Such matrices can be plotted as contour plots, as shown in Figure 2, and they reflect the presence of (and interferences between) several compounds.

Fluorescence spectroscopy is sensitive to fluorophores present in MBR systems and to mutual interferences of these compounds with their surrounding media [22]. Therefore, fluorescence spectra obtained from complex systems capture extensive information from the media, not only regarding the natural fluorophores present, but also related to the optical characteristics of the media (such as turbidity or color) that impact the path of the light, due to quenching effects promoted by the presence of interfering compounds (which can be either fluorescent or nonfluorescent). In fact, destructive interferences are quite common in complex systems and can be caused by a large diversity of phenomena, such as the superimposition of emission spectra from different fluorescent species, quenching effects, and inner filter effects (including the re-absorbance of the light emitted by fluorophores and light scattering due to the presence of suspended solids or turbidity) [22,23].

The presence of interferences in fluorescence spectra from complex media is usually overcome by using different mathematical methodologies to deconvolute spectral peaks and extract the meaningful information from the fluorescence spectra. Actually, interferences can also represent an advantage over other analytical methodologies, since they are also a source of information about the status of the system (as discussed below in Section 2.2 and Section 2.3). 

Two-dimensional fluorescence spectroscopy has been studied for monitoring wastewater treatment plants (industrial and domestic, conventional, and MBR plants), to monitor the presence of dissolved organic matter in rivers and water supply, and to monitor organic fouling in membrane processes.

In MBRs, 2D fluorescence spectroscopy is used to characterize organic matter and foulants, mostly to detect the nature of compounds, or to perform some semi-quantitative analysis through comparison of spectra peaks. Due to the presence of several interferences, 2D fluorescence spectroscopy has only been applied directly to characterize fractions collected from MBRs, which were filtered and/or diluted [12,24,25,26,27,28]. In fact, although the spectra obtained by 2D fluorescence spectroscopy are useful to identify the presence of some natural fluorophores (e.g., proteins and humic-like substances), the direct use of fluorescence intensity in one region of excitation/emission wavelengths cannot be correlated directly with the concentration of fluorophores in standard solutions, due to the several fluorescence interferences in the biological media. Therefore, to extract reliable information (both qualitative and quantitative) from fluorescence EEMs, different strategies have been applied on the basis of sample preparation and/or mathematical methods for spectral deconvolution. 

Some strategies commonly applied to overcome fluorescence interferences in MBRs usually involve dilution and acidification of samples [29] and the use of inner-filter corrections based on absorbance spectroscopy [30]. Some studies assessed ratios between peaks and compared them across different MBRs or streams to assess the changes in composition [31], as well as calculate removal percentages related to identifiable peaks (protein-like, humic-like, and microbial-derived peaks) [12]. Other studies involved more calculations, such as the estimation of fluorescence regional integration (FRI), and used 2D fluorescence to quantify differences of dissolved organic matter within different samples [32,33,34,35,36]. EEMs with FRI were used to characterize different EPS extracts obtained from membrane backwash (using different solutions) when characterizing MBRs for the treatment of an industrial wastewater [35].

Parallel factor analysis (PARAFAC), a mathematical algorithm based on principal component analysis (PCA), was developed to deconvolute spectral data such as excitation/emission matrices [37], and it is increasingly being applied to characterize the fluorescence properties of dissolved organic matter [38]. In fact, the utilization of fluorescence spectroscopy was previously reviewed as a monitoring tool for wastewaters [39]. In this review, the use of fluorescence for characterization of dissolved organic matter in wastewaters is detailed, and more information about the use of PARAFAC is given for the deconvolution of fluorescence peaks and identification of fluorophores.

In MBRs, Xue et al. used fluorescence EEMs to assess dissolved organic matter and compare the performance of different bioreactors [40]. In this study fluorescence spectra were analyzed both through FRI and PARAFAC. Although PARAFAC allowed qualitatively characterizing the streams, the authors used FRI to calculate and compare removal rates relative to five different spectral regions. The impact of online chemical cleaning in MBRs was also previously assessed by 2D fluorescence spectroscopy combined with PARAFAC [41].

As also noted by Stedmon and Bro, even after application of a mathematical tool such as PARAFAC, the interpretation of fluorescence EEMs cannot be achieved directly [38]. Differences in fluorescence intensity between different compounds do not mean that one compound is present in a higher concentration than the other, since the fluorescence signal results from compound concentration, molar absorptivity, and quantum efficiency. Therefore, the use of ratios is most appropriate to characterize quantitative differences between samples. 

The use of fluorescence EEMs for characterization of dissolved organic matter in MBRs was also reviewed in 2020, and the same interference effects were pointed out as limiting the application of 2D fluorescence spectroscopy, especially when linear addition or linear dependence based on fluorescence peaks is used for quantification purposes [42]. In one study, the volumes of fluorescence (similar to FRI calculation) were used in linear correlational models with the results of size-exclusion liquid chromatography combined with organic carbon and organic nitrogen detection (LC–OCD/OND) [43]. Although tendencies were found between these data, no correlations were found with proteins and polysaccharides assessed by colorimetric methods, and different organic matter composition had different correlations with the fluorescence data; thus, new calibrations should be performed before using the technique as a pseudo-quantitative method. 

As mentioned before, fluorescence spectroscopy captures several characteristics from the media, not only regarding the natural fluorophores present, but also related to the optical characteristics of the media (such as turbidity or color) that impact the path of the light, and due to quenching effects promoted by the presence of interfering compounds. Despite the difficulty in overcoming these interferences, they are also a source of information about the complex media; thus, the fluorescence spectra (EEMs) can be seen as fingerprints reflecting the status of the system. The matrices obtained by 2D fluorescence spectroscopy encode information concerning not only the presence of natural fluorophores in the system [44], but also their interactions with the involving media [45]. Additionally, since the fluorescence response is sensitive to the environmental conditions (pH, ionic strength, and salt composition), fluorescence EEMs can also capture information about the performance of biological systems operated under specific process environments. This ability represents an advantage over other analytical methodologies that have been applied so far for MBR monitoring. Nevertheless, such information must be disclosed using adequate mathematic tools.

### 2.2. Extraction of Information from Fluorescence Data

To rapidly extract the full contextual information contained in spectroscopic data, through the last years, several authors have suggested different non-mechanistic approaches [21,37,46,47,48]. Non-mechanistic models, based on machine learning, can correlate large sets of data (including 2D fluorescence spectra and other parameters) extracting hidden information and disclosing nonobvious relationships between different parameters.

As summarized in Table 1, the extraction of information from 2D fluorescence spectroscopy and its first use as an online multiparameter monitoring tool for biological processes was proposed in the late 1990s, through the mathematical selection of different excitation/emission wavelengths that mostly correlated with different process parameters [44,49,50,51]. At the same time, specific user-friendly mathematical tools able to deconvolute large spectroscopic data were also developed, such as PARAFAC [37], enabling an easier use of 2D fluorescence spectroscopy and a better understanding of fluorescence regions (where different fluorophores are present). However, the first use of entire EEMs, without fractionation of the spectra, was proposed later (2001) for applied to the monitoring of an extractive membrane bioreactor [21,52]. Instead of removing interferences from fluorescence EEMs and correlate peaks with the concentration of specific compounds, these studies used an approach based on pattern recognition, (artificial neural networks (ANN)) to deconvolute entire fluorescence matrices from an MBR using a mixed microbial culture.

Principal component analysis (PCA), including PARAFAC (discussed in the previous section), is often used to deconvolute the peak resolution of spectra or as a compression tool to reduce the dimension and data redundancy of fluorescence EEMs, and eliminate noise, prior to projection to latent structures (PLS) modeling [46] or to feed ANN [53]. In fact, principal component analysis (PCA), projection to latent structures (PLS) regression, and artificial neural networks (ANNs) are among the mostly used data-mining techniques used for spectral deconvolution [47].

PCA algorithms are unsupervised machine learning tools that replace the representation of objects from their initial space into a new coordinate space (principal components) with reduced noise and lower dimensionality. PCA allows obtaining the score matrix, where the initial data are represented in the new reduced coordinate system, and the loading matrix, which describe the ‘distance’ between the initial coordinate system and principal component coordinate system. Therefore, PCA can be used as a qualitative tool through the analysis of scores and loadings plots, where differences and similarities between samples (or observations) and between variables can be assessed visually. Additionally, PCA can be used to compress the number of parameters needed to describe spectroscopic data while eliminating noise (the score matrix). The score matrix includes the most relevant information from original data with reduced dimension; thus, it can be more easily correlated with analytic and process data. Scores can be correlated with analytic and performance data via either univariate correlations or multivariate modeling, where compressed fluorescence data can be combined with process data to monitor one or several parameters simultaneously.

**Figure 3:**
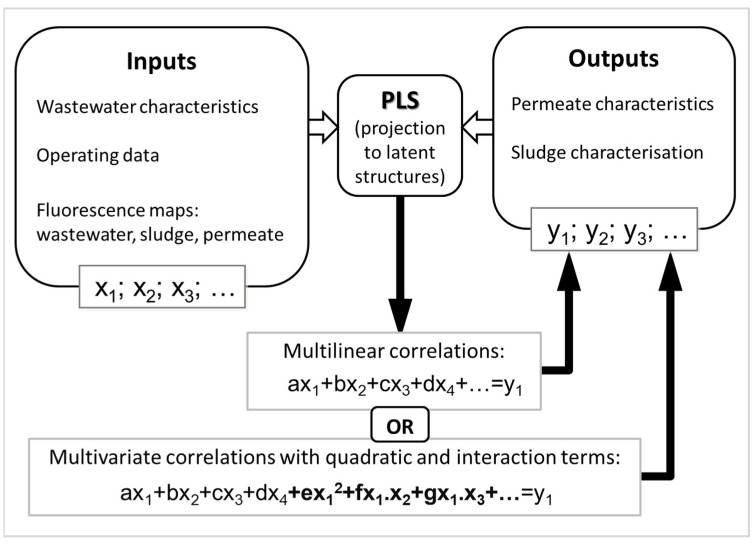
Schematic representation of PLS modeling methodology applied to MBR monitoring.

Multilinear regression can be achieved through projection to latent structures (PLS) to reveal relationships between datasets. PLS modeling maximizes the covariance between the input matrix X and the output Y, aiming at the prediction of dependent variables by iteratively decomposing the X and Y matrices into reduced orthogonal factors. PLS is a simple but powerful predictive multilinear modeling technique due to its ability to handle collinearity among variables, noise, and missing data [60]. Using multilinear regression, it is possible to correlate the information contained in fluorescence EEMs, directly or after PCA compression, with a quantitative output parameter. After a first stage of calibration, where the correlation between the datasets is stablished, it is possible to extract quantitative information from the fluorescence spectra (Figure 3). Furthermore, to account for nonlinear interactions, quadratic and interaction terms of input data parameters can be incorporated in PLS modeling [46,55], as shown in Figure 3.

In the situations where the nonlinearity between input fluorescence data and the output is accentuated, nonlinear techniques such as artificial neural networks (ANNs) have been employed to correlate fluorescence data with process parameters [21]. Artificial neural networks are, as multivariate regressions, supervised mathematical tools, meaning that they can ‘learn’ how the inputs are related to the outputs. ANN mimics the processing of information of the human brain, based on pattern recognition, and it can correlate data through linear or nonlinear weighted functions. As in PLS modeling, the network is first trained (calibrated) with both input and output data of the training set, before it can be used. The reduction in input nodes to the ANN can also be achieved using PCA to compress fluorescence data [53].

While ANNs can disclose more complex interactions among data, through complex nonlinear correlations, PLS models result in mathematical equations (either linear or nonlinear) that are simpler and easier to apply and interpret (through the weight of inputs).

Models based on operating conditions, streams characteristics, and biological reactions are essential to translate the data into the performance parameters that are used for monitoring MBR systems [61]. In particular, these machine learning tools allow achieving process monitoring based on available data and can incorporate different types of data in the same tool (e.g., operating conditions, spectroscopic data, monitoring parameters), increasing the quality of the models achieved and allowing the development of control tools [62]. 

### 2.3. Machine Learning and 2D Fluorescence Spectroscopy for MBR Monitoring

Fluorescence EEMs and an ANN algorithm were used to estimate the concentration of 1,2-dichloroethane, ammonia, and chloride in the medium of an extractive MBR used for the degradation of chlorinated organic compounds [21]. In this study, the fluorescence spectra were collected in various places of the membrane surface, where a biofilm was developing, and a previous assessment based on spectral subtraction concluded that a nonlinear technique (as ANN) would be required to extract the useful information they contained. 

In another study, PLS modeling was used to correlate 2D fluorescence data with the wastewater treatment performance data of an MBR operated for the treatment of domestic wastewater [54]. In this study, EEMs obtained from the influent and from the permeate streams were correlated with influent and effluent total chemical oxygen demand (COD), respectively. Despite the good results obtained for COD, this approach was still not sufficient to assess other performance parameters of the MBR system, such as influent and effluent ammonia and phosphorus. The entire excitation/emission matrices (EEM) were used directly in PLS modeling, allowing the examination of the regression coefficients of the PLS models obtained; thus, the 2D fluorescence spectral regions that were strongly correlated with the COD contents were identified. In addition to humic-like and protein-like regions, this study showed that light scattering (which is often due to media turbidity, suspended solids, and high solute concentrations) was also determinant in COD prediction, for both the influent wastewater and the permeate. These results suggest that a pretreatment of fluorescence data that removes scatter would not be appropriate. The information in fluorescence EEMs resulting from light interference can carry additional information about the system status and, thus, be essential to predict performance parameters in an MBR. On the other hand, other mathematic pretreatments, such as PCA, can be helpful, not only to reduce data dimension prior to feed a correlation model, but also to select spectral regions of higher variability. 

The application of PCA to fluorescence EEMs prior to ANN modeling was performed for monitoring an extractive MBR, resulting in a reduction in the number of inputs (i.e., fewer input nodes in the ANN algorithm) and, thus, reducing the computational effort (i.e., less time for required for model learning) [53].

The relationships between operating parameters and performance variables in MBRs are complex and interdependent; therefore, a combined approach using fluorescence data and process parameters as inputs was also explored for MBR monitoring [46,52]. To improve prediction and allow control of the process, fluorescence EEMs obtained from an extractive MBR were also combined with a current and historic process operation using ANN modeling to predict seven different process parameters [52]. This study showed that current and past operating conditions are highly relevant for the overall performance of the system, enabling better prediction of the performance and facilitating further implementation of the control tools.

**Figure 4:**
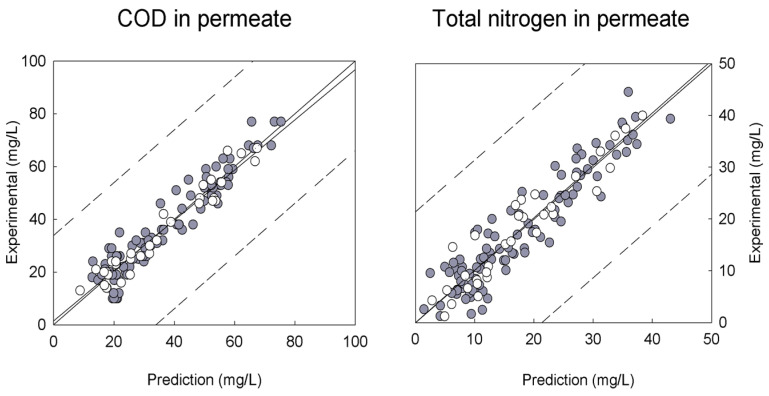
Fitting plots of experimental versus modeled COD and nitrogen concentrations in the permeate of an MBR. The training dataset is represented as gray circles, and the validation dataset used in PLS modeling is represented as open circles.

In an MBR for wastewater treatment, fluorescence EEMs, after compression with PCA, operating and analytical data were modeled through PLS to describe transmembrane pressure (TMP), effluent quality (total COD, soluble COD, nitrite plus nitrate concentration, total nitrogen and total phosphorus in the permeate) and the biomass concentration in the bioreactor (MLSS). In this study, the correlations between inputs and outputs were obtained not only through multilinear PLS modeling (TMP, and soluble and total COD) but also through the incorporation of quadratic and interaction terms of the compressed fluorescence matrices in PLS modeling (nitrite and nitrate concentration, total nitrogen, total phosphorus, and MLSS). The predictive potential of these models is illustrated in Figure 4, depicting the fitting of the PLS models developed to predict total COD and total nitrogen in the permeate of an MBR. The model for estimation of COD uses information extracted from fluorescence EEMs combined with the TMP assessed online, while the estimation of nitrogen is achieved with fluorescence EEMs combined with temperature.

In addition to the correlational tools, the mathematical selection of the most useful inputs is highly relevant for the extraction of information from fluorescence EEMs, and when combining different types of parameters as inputs. The selection of inputs is essential in the optimization of PLS models resulting in better prediction, lower errors, and simpler equations with fewer inputs [46]. Therefore, it is possible to obtain more information about the correlations between the output and the inputs. 

The applicability of 2D fluorescence spectroscopy and machine learning to simultaneously monitor multiple key MBR performance parameters with minimal analytical effort has, thus, been fully demonstrated in the literature.

In another study, a different approach was used to maximize the potential of using 2D fluorescence spectroscopy for MBR monitoring using hybrid models, developed through the integration of mechanistic and PLS models [55]. In this modeling approach, a parametric model used to predict biological performance of wastewater treatment systems (activated sludge model number 3—ASM3) was complemented by PLS modeling to predict the residuals of the mechanistic model. This modelling strategy was used to improve the prediction ability of an easy-to-implement mechanistic model, with minimal additional monitoring effort, taking advantage of the ability of PLS to combine different types of parameters. Hybrid models were developed to predict three MBR performance parameters: MLSS, COD in the permeate, and nitrite and nitrate concentration in the permeate. 

Through all studies developed so far, 2D fluorescence spectroscopy was shown to be a powerful monitoring tool for application not only in MBRs for domestic wastewater treatment, but also for other biological systems and other membrane processes involving organic compounds, since fluorescence matrices proved to be well related to these compounds through adequate mathematical tools. 

Other studies in membrane processes involving biological and/or organic compounds showed that fluorescence spectroscopy also has great potential for monitoring fouling development [56,57,58,59]. In these studies, fluorescence EEMs obtained on membrane surfaces were used to assess not only fouling but also the effect of cleaning at the membrane surface.

## 3. Conclusions and Future Perspectives

Most studies using 2D fluorescence spectroscopy in MBRs focused on the characterization of organic matter, while only a few of them studied the use of fluorescence as a multiparametric monitoring tool to be applied in real time. Nevertheless, different strategies have already been developed and applied to use the information from fluorescence EEMs for characterization and monitoring purposes in MBRs. 

While the applicability of 2D fluorescence spectroscopy as a monitoring and characterizing tool in MBRs is still limited, the use of fluorescence EEMs is being successfully studied for monitoring other biological and membrane processes, e.g., for monitoring specific compounds and characterization of fouled membrane surfaces. Therefore, in view of the specific needs of MBRs in terms of monitoring and control, there is still a large range of possibilities of application of fluorescence and machine learning tools that can be explored for MBRs to achieve a multiparameter monitoring and control tool.

The use of 2D fluorescence spectroscopy as a monitoring tool in MBRs was proven to have several advantages:

- Using an optical probe, it is possible to collect fluorescence EEMs either from liquid media or from membrane surfaces.
- It does not consume reagents, and it can be applied online and without disturbing the system.
- As a fingerprinting technique, the use of EEMs enables characterizing the status of the system and can be used as a multiparameter tool with reduced analytical effort.
- After the initial establishment of the multivariate statistical models for a given process, the application of machine learning to fluorescence enables the continuous update and improvement of models with new process data (extending the domain of applicability).

Despite the high potential of fluorescence spectroscopy to monitor MBRs, more work is still needed to increase the number of monitoring parameters and to develop software (mathematical tools or machine learning) for data interpretation and usage.

- The mathematical multivariate approaches followed so far can be used to explore further information contained in fluorescence spectra to predict additional performance parameters.
- The acquisition of fluorescence spectra at the membrane surface, in situ, should also be considered in future research work.
- Machine learning can be used to integrate different monitoring and operating parameters in user-friendly monitoring systems (which translate the monitoring data into performance parameters that can be designed to support operating decisions) and implement automatic control.
- The implementation of such a monitoring (and control) tool requires a simple, robust, and economic spectrofluorometer equipped with optical probes and the use of an optical switchbox for monitoring at multiple locations of the system.
- Above all, the development of a dedicated and user-friendly software is essential to integrate the acquisition of fluorescence data, other data sources, and mathematical tools.

After implementation of data acquisition and software for data interpretation, the use of 2D fluorescence spectroscopy requires neither reagents nor other equipment, outside of a spectrofluorometer equipped with an optical probe and a computer. Furthermore, even when a large EEM is acquired, the spectral acquisition can be performed in less than 15 min, providing results with high frequency when installed in situ. Therefore, by using 2D fluorescence spectroscopy as a multiparametric monitoring tool, the costs for monitoring can be reduced while the frequency of monitoring can be increased (when compared with conventional, offline analysis), thereby providing information for process control and performance optimization (leading to improved process efficiency and stability). 

In view of all the work so far using 2D fluorescence spectroscopy, this method is without any doubt highly relevant for the characterization and online monitoring of MBR systems. However, the potential of the application of machine learning to extract meaningful information and correlate it with performance parameters (as well as to develop warning systems and automatic control tools) is still far from fully explored.

## Figures and Tables

**Table 1:** Summary of studies using 2D fluorescence spectroscopy deconvoluted with mathematical methods for process characterization and monitoring.

Process	Part of Spectra	Sample Preparation	Data Interpretation	Inputs	Outputs	Year	Ref
Microorganism cultivation	Selected regions	No	Selection of Ex/Em pairs combined with multivariate data analysis	Selected pairs of Ex/Em	Several performance parameters (cell concentration, medium composition, turbidity)	1996 to 1998	[44,49,50,51]
Extractive MBR	Entire EEM	No	ANN	Entire EEMs	Outlet concentration of 1,2-dichloroethane (pollutant); ammonia; chloride	2001	[21]
Extractive MBR	Entire EEM	No	ANN	Operational parameters (present and past) + entire EEMs	Seven performance parameters	2005	[52]
Extractive MBR	Entire EEM	No	PCA + ANN	Process performance data + principal components of EEMs	Seven performance parameters	2007	[53]
MBR	Entire EEM	No	PCA + PLS regression	Principal components of EEMs	COD in permeate	2011	[45]
MBR	Entire EEM	No	PLS regression	Entire EEMs	COD in feed; COD in permeate	2011	[54]
MBR	Entire EEM	No	PCA + PLS + input selection	Principal components of EEMs; additional monitoring parameters	Seven performance parameters	2012	[46]
MBR	Entire EEM	No	Mechanistic modeling + PLS regression with PCA of EEMs	Characterization parameters + principal components of EEMs	MLSS; COD in permeate; NO_2_ + NO_3_ in permeate	2013	[55]
Reverse electrodialysis	Entire EEM	Directly at membrane surface	PCA + PLS regression	Operating data + principal components of EEMs	Pressure drop; stack electric resistance; net power density	2015	[56]
Reverse electrodialysis	Entire EEM	Directly at membrane surface	PCA	Principal components of EEMs	Qualitative analysis of membranes surface	2016	[57]
MBR	Specific peaks	No	PARAFAC	PARAFAC components	Qualitative analysis of PARAFAC components	2017	[41]
MBR	Regions	Dilution	FRI	Volume of fluorescence from EEMs regions	Protein-like and humic-like substances	2017	[43]
MBR and other	Regions; peaks	No	FRI; PARAFAC	PARAFAC components	Qualitative analysis of PARAFAC components	2022	[40]
Anion-exchange MBR	Entire EEM	Directly at membrane surface	PCA	Principal components of EEMs	Qualitative analysis of membranes surface	2022	[58]
Nanofiltration	Entire EEM	Directly at membrane surface	PCA	Principal components of EEMs	Qualitative analysis of membranes surface	2023	[59]

ANN—artificial neural networks; COD—chemical oxygen demand; FRI—fluorescence regional integration; MLSS—mixed liquor suspended solids; PARAFAC—parallel factor analysis; PCA—principal component analysis; PLS—projection to latent structures.

## Data Availability

Not applicable.
